# STAT3 Signaling in Breast Cancer: Multicellular Actions and Therapeutic Potential

**DOI:** 10.3390/cancers14020429

**Published:** 2022-01-15

**Authors:** Sarah Q. To, Rhynelle S. Dmello, Anna K. Richards, Matthias Ernst, Ashwini L. Chand

**Affiliations:** Olivia Newton-John Cancer Research Institute, School of Cancer Medicine, La Trobe University, Heidelberg, VIC 3084, Australia; Sarah.Bennett@onjcri.org.au (S.Q.T.); Rhynelle.Dmello@onjcri.org.au (R.S.D.); Anna.Richards@onjcri.org.au (A.K.R.); Matthias.Ernst@onjcri.org.au (M.E.)

**Keywords:** STAT3, breast cancer, IL-6, IL-11, metastasis, targeted therapies

## Abstract

**Simple Summary:**

Many signaling pathways are overactive in breast cancer, and among them is the STAT3 signaling pathway. STAT3 is activated by secreted factors within the breast tumor, many of which are elevated and correlate to advanced disease and poor survival outcomes. This review examines how STAT3 signaling is activated in breast cancer by the proinflammatory, gp130 cytokines, interleukins 6 and 11. We evaluate how this signaling cascade functions in the various cells of the tumor microenvironment to drive disease progression and metastasis. We discuss how our understanding of these processes may lead to the development of novel therapeutics to tackle advanced disease.

**Abstract:**

Interleukin (IL)-6 family cytokines, such as IL-6 and IL-11, are defined by the shared use of the gp130 receptor for the downstream activation of STAT3 signaling and the activation of genes which contribute to the “hallmarks of cancer”, including proliferation, survival, invasion and metastasis. Increased expression of these cytokines, or the ligand-specific receptors IL-6R and IL-11RA, in breast tumors positively correlate to disease progression and poorer patient outcome. In this review, we examine evidence from pre-clinical studies that correlate enhanced IL-6 and IL-11 mediated gp130/STAT3 signaling to the progression of breast cancer. Key processes by which the IL-6 family cytokines contribute to the heterogeneous nature of breast cancer, immune evasion and metastatic potential, are discussed. We examine the latest research into the therapeutic targeting of IL-6 family cytokines that inhibit STAT3 transcriptional activity as a potential breast cancer treatment, including current clinical trials. The importance of the IL-6 family of cytokines in cellular processes that promote the development and progression of breast cancer warrants further understanding of the molecular basis for its actions to help guide the development of future therapeutic targets.

## 1. Introduction

**Figure 1:**
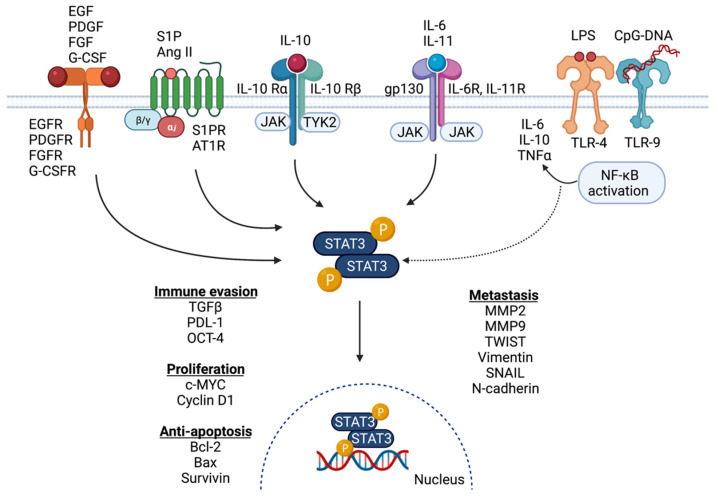
Activation, signaling partners and downstream targets of STAT3 in breast cancer. STAT3 is activated by cytokine signaling when IL-6 or IL-11 ligands bind to a hexameric receptor complex consisting of their specific receptor and gp130. This leads to a recruitment of JAK kinases to phosphorylate and activate STAT3, which dimerizes and translocates into the nucleus. Together with NF-κB and GLI1, STAT3 activates transcription of a unique set of target genes which promote metastasis immune evasion, proliferation and resistance to apoptosis. Figure generated in Biorender (Available online: Biorender.com (accessed on 8 December 2021)).

The IL-6 superfamily of cytokines comprises nine secreted ligands: Interleukin-6 (IL-6); Interleukin-11 (IL-11); leukemia inhibitory factor (LIF); oncostatin M (OSM); ciliary neurotrophic factor (CNTF); cardiotrophin-1 (CT-1); cardiotrophin-like cytokine (CLC); interleukin-27 (IL-27); and interleukin-31 (IL31) [1]. Whilst each of these ligands binds to a specific transmembrane receptor, referred to as the receptor α chain, the IL-6 family of cytokines are classed together for their shared use of the ubiquitously expressed transmembrane glycoprotein-130 beta subunit (gp130) [2]. These cytokines have wide-ranging biological activities in different cell types, including hematopoiesis, neuronal regeneration, bone remodeling, inflammation and immune response, as well as many cancer processes [3]. Specifically, IL-6, IL-11, their receptors and downstream transcription factor STAT3, are known to be highly expressed in breast cancer (Figure 1), and the role of cytokine-induced STAT3 signaling in the breast tumor microenvironment is multifaceted [4].

The signal transducers and activators of transcription (STAT) family of transcription factors are a group of seven structurally similar, highly conserved proteins comprising STAT1, STAT2, STAT3, STAT4, STAT5a, STAT5b and STAT6 [5]. Through their common functional domains, the STAT transcription factors bind to DNA sequences and mediate transcription of a host of target genes required for normal physiologic function. However, dysregulation attributed to STATs has also been implicated in a number of cancer types, including breast cancer [6]. Of these, STAT3, in particular, has been studied for its role in cancer progression, proliferation, metastasis, modulation of tumor-related immune responses and therapeutic resistance. Particular attention has been drawn to not only to STAT3 hyperactivity in cancer, but the increased abundance of its activating cytokines, the IL-6 superfamily of cytokines. This review will focus on the role of IL-6 and IL-11, in particular, in driving increased STAT3 transcriptional activity in breast cancer, and how this leads to metastasis and immune evasion within the tumor microenvironment. We will examine some of the transcriptional networks activated by STAT3 downstream of IL-6 and IL-11 and review the progress that has been made to therapeutically target this signaling axis.

## 2. Activation of STAT3 in the Breast Cancer Microenvironment

### 2.1. Activation of STAT3 Signaling in Breast Cancer

The multifaceted potential of STAT3 as an oncogenic transcription factor has been widely studied and recognized by its role in regulating the expression of genes related to cancer cell proliferation, invasion, migration, anti-apoptosis, immunosuppression, stem cell regeneration and autophagy [3,4,7]. Although the IL-6 family of cytokines are major players in the upstream activation of STAT3 signaling, emerging evidence has identified a number of important activators of this signaling pathway. Traditionally, STAT3 signaling activation was thought to be limited to cytokines and growth factors including IL-6 and IL-11, IL-10, IL-23 and their receptors, as well as EGFR, PDGFR, FGFR, leptin receptor, G-CSFR and CNTF [3,8]. Recently, Toll-like receptors (TLRs) including TLR-9 and TLR-4, as well as G-protein coupled receptors (GPCRs) including S1PR and AT1R, have also been implicated as important STAT3 signaling activators (Figure 1) [3,9,10,11,12,13,14,15].

The hyperactivation of IL-6 and IL-1-dependent STAT3 signaling is correlated with poor patient prognosis and occurs in most human cancers including breast cancer [16]. The pro-inflammatory cytokines IL-6 and IL-11 are produced by multiple cell types within the tumor microenvironment, such as tumor-infiltrating immune cells, stromal cells and tumor epithelial cells. These cell types produce cytokines which then act in an autocrine or paracrine fashion to activate the STAT3-dependent gene transcription [1,16,17,18]. Additionally, IL-6 plays a role in the recruitment of immune cells within the tumor microenvironment, thereby stimulating the production of other pro-inflammatory cytokines, including IL-1β, IL-8, and TNFα, thus providing a link between the inflammatory process and tumor progression (Figure 1) [19,20].

Classical STAT3 signaling is activated by the binding of IL-6 and IL-11 to their respective cell surface receptors IL-6R and IL-11R, and the interaction of this complex to the shared cognate receptor gp130 (also known as IL-6Rβ) to activate intracellular STAT3 signaling [4,16]. In addition, IL-6 and IL-11 are also able to bind to the secreted form of their respective receptors sIL-6R and sIL-11R, to activate trans-signaling, following the binding of this complex with gp130 [16,21,22]. Although IL-6R and IL-11R are expressed in specific cell types, gp130 is ubiquitously expressed allowing for the activation of intracellular STAT3 signaling in cells with limited or no IL-6R and IL-11R expression, via sIL-6R and sIL-11R by tumor infiltrating neutrophils, monocytes and T-cells [16,21,22,23]. The soluble form of gp130 (sgp130) acts as a negative regulator of trans-signaling by competing with membrane-bound gp130 to bind to the ligand–receptor complex, thus preventing the activation of intracellular STAT3 [16,23]. Intracellular STAT3 activation is triggered by the hetero–hexameric complex, which consists of IL-6, IL-6R and gp130 or IL-11, IL-11R and gp130, which interacts with JAK1/JAK2 resulting in phosphorylation of gp130 at several tyrosine residues, that act as docking sites for STAT3 [4]. Once STAT3 binds to gp130, it is phosphorylated by JAK proteins resulting in STAT3 activation, dimerization and translocation into the nucleus thus enabling STAT3 mediated transcription of target genes [4,24]. The suppressor of cytokine signaling 3 (SOCS3), which inhibits JAK protein activity, is one of the target genes mediated by STAT3 transcription and tightly regulates STAT3 signaling by maintaining a negative feedback loop [16,24,25]. Negative regulation of this pathway is also maintained by several tyrosine phosphatases, endogenous proteins that promote STAT3 degradation and oncogenic and cellular miRNAs [26,27,28].

### 2.2. Signaling Partners and Downstream Targets of STAT3 in Breast Cancer

As a transcription factor, STAT3 regulates the expression levels of many target genes in a normal physiological and cancerous cellular environment (Figure 1). Changes in STAT3 signaling can cause the upregulation of cancer-driving oncogenes, such as c-MYC and VEGF, or the downregulation or a silencing of tumor-suppressor genes including PTEN, p53 and PTPN6 [3,29], resulting in signaling favorable to tumor development and progression. An understanding of specific downstream targets of STAT3 will enable an elucidation of the specific mechanisms that action STAT3 has in cancer progression. Changes in cellular gene expression due to STAT3 can influence the ability to invade, metastasize, proliferate, evade the immune response, and resist cell death signals [30]. In normal cellular function, the activity of STAT3 is tightly regulated by the suppressor of cytokine-signaling 3 protein (SOCS3), which is rapidly induced via STAT3 binding to its promoter to sequester activation of STAT3 in a negative feedback mechanism [31]. This feedback is often dysregulated in tumor epithelial cells and the immune architecture of the tumor microenvironment, leading to excessive STAT3 signaling during tumor development and progression [32]. STAT3 is also involved in positive feedback loops which involve the regulation of gene sets that, in turn, stimulate STAT3 activity. For example, STAT3 directly regulates NF-κB expression [33], and STAT3 also requires NF-κB for its recruitment to the promotor of the fascin gene, FSCN1, to form an active transcriptional complex [34]. The increased expression of FSCN1, an actin-binding protein, leads to cellular reshaping with increased actin-based cell protrusion formation, and is a phenotypic feature leading to decreased cell adhesion and increased motility in aggressive breast cancer subtypes, particularly triple negative breast cancer (TNBC) [35].

The activation of STAT3 via its phosphorylation at Tyrosine 705 residue is a critical juncture in its downstream regulation of target genes. STAT3 is a central node in several signaling pathways, such as IL-6/IL-11 [36], sonic hedgehog [37], and ERK signaling [38], and the crosstalk between these regulatory relationships highlights essential, pro-tumorigenic roles. Following its phosphorylation, dimerization and translocation into the nucleus, STAT3 binds to a consensus-binding sequence in the promoter region of target genes to regulate their transcriptional activity [39,40]. Utilizing genome-wide chromatin immunoprecipitation-sequencing (ChIP-seq) in the immortalized mammary epithelial cell line MCF-10A, STAT3 binding sites were found to be enriched in the promoters of genes regulating cell movement, growth proliferation and inflammation in a normal breast setting [33]. A complementary study has also examined STAT3 binding sites in TNBC cell lines and patient tumors by ChIP-seq, finding binding enrichment for pathways involved in extracellular matrix organization, extracellular structure organization, collagen metabolic processes, anchoring junctions, adherens junctions and the regulation of locomotion [41]. These findings heavily implied a significant role for STAT3 in cellular invasion and metastasis in TNBC development.

The co-overexpression and physical interaction of phospho-STAT3 and GLI1, the glioma-associated oncogene, is found in approximately 60–70% of both TNBC and HER2-enriched breast cancers [42]. Co-overexpression of these proteins was also present in lymph node metastases, and patients with co-activated STAT3/GLI1 breast tumors had poor long-term survival outcomes [42,43]. This relationship is also established in non-small cell lung cancer and chronic lymphocytic leukemia [37,44]. ChIP-seq analysis identified genes co-activated by STAT3 and GLI1 by analyzing overlapping chromatin-binding signatures. Three gene were found to be significantly upregulated with the co-overexpression of STAT3/GLI1, R-Ras2, Cep70 and UPF3A, which are known to play significant roles in PI3K signaling and microtubule disorganization [45,46]. The high expression of these genes is associated particularly with reduced metastasis-free survival in TNBC and HER2-enriched breast cancer, a trend which has also been observed in pancreatic and colon cancer [42,45,46,47].

**Figure 2:**
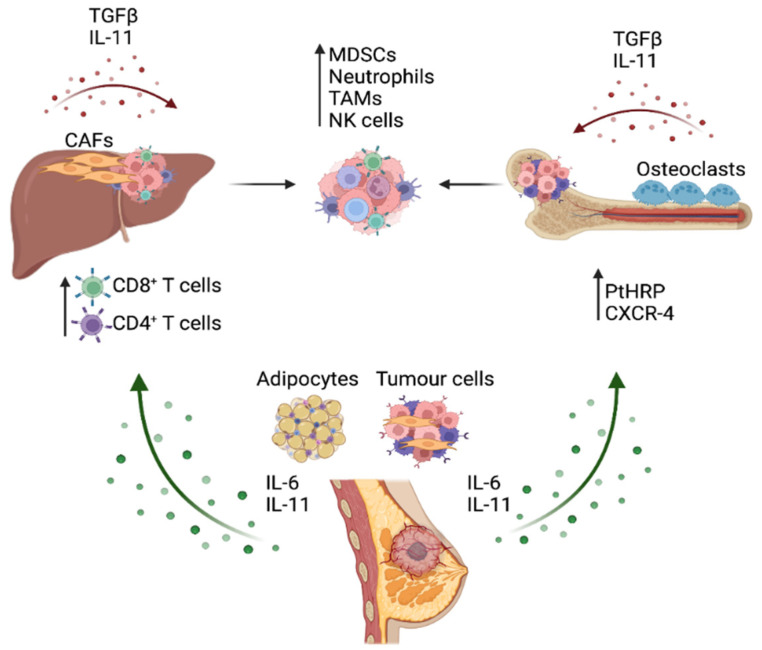
The involvement of IL-6 and IL-11 in the metastatic process in breast cancer. Cellular sources of IL-6 and IL-11 within the tumor microenvironment of the primary tumor in the breast and distal organ sites are illustrated. The excessive secretion of IL-6 and IL-11 support breast cancer development and enable the outgrowth of metastatic lesions in distal sites by promoting either the recruitment or reprogramming of immune cell types. Figure generated in Biorender (Available online: Biorender.com (accessed on 8 December 2021)).

The known interaction of STAT3 with other transcription factors and crosstalk with other signaling pathways provides the potential for investigating combination targeted therapies. One example is research into the combination of STAT3 inhibition with an immune checkpoint blockade. STAT3 is known to regulate many genes involved in immune escape and chemoresistance, such as TGF-β [48], VEGF [49], NF-κB [34], OCT-4 and c-MYC [50], presenting an attractive option for dual-targeting approaches. Recently, a preclinical study evaluated the combined effects of an anti-PD-1 antibody with a STAT3 inhibitor (STX-0119) in an in vivo pancreatic cancer model; however, no significant anti-tumor benefits were observed and the study was terminated due to safety concerns from excessive weight loss [51]. However, the synergistic combination of anti-PD-1 therapy and the natural STAT3-inhibiting compound curcumin augmented anti-tumor responses in a colon carcinoma model [52]. This is potentially due to the broad range of therapeutic targets touted as downstream of curcumin, including STAT3, NF-κB, prostaglandin E2, COX-2 and TGF-β amongst many others [53]. It is therefore difficult to determine if the anti-cancer effects observed when anti-PD-1 therapy was combined with STAT3-targeting curcumin was specific to STAT3-mediated effects or if other pathways were involved. Although further studies involving the assessment of STAT3 inhibition in combination with immunotherapies are needed, the comprehensive crosstalk between STAT3-dependent pathways and immune checkpoints presents an attractive therapeutic target in breast cancer (Figure 2).

### 2.3. STAT3 Signaling and Patient Outcome

Elevated expression or gene amplification of STAT3 and its upstream activators may be used as a prognostic indicator in breast cancer. Breast tumors with high expression of the IL-11 ligand are correlated with, higher histological grade, local or distant recurrence and poorer long-term survival outcomes [54]. Serum or intra-tumoral levels of IL-11 are highest in patients developed bone metastases and who have shorter disease-free survival periods [55], however similar correlations have not been observed for transcripts or protein expression of the IL-11 receptor [1,56]. Interestingly, an analysis of three independent cohorts NETHERLANDS, METABRIC and OSLOVAL concluded that JAK2 mRNA expression was protective in all types of breast cancer. Favorable prognosis and long survival were characteristic of patients with high JAK2, however poor correlation between mRNA and protein levels in their studies led the authors to postulate that mRNA levels alone may not be a reliable prognostic indicator [57]. There is conflicting evidence regarding the prognostic value of STAT3 mRNA expression or immunostaining of phosphorylated STAT3 (phospho-STAT3), marking transcriptionally active STAT3. Positive phospho-STAT3 (Y705) expression is highest in TNBC, followed by luminal A and luminal B, with HER2-enriched tumors showing the lowest rate of phospho-STAT3 immunostaining [58], suggesting that phospho-STAT3 may be a hallmark of more aggressive breast cancer subtypes. Other studies and meta-analyses have positively correlated nuclear phospho-STAT3 localization to highly differentiated tumors, higher tumor grade and lymphatic metastasis, though there appears to be no relationship to overall survival outcomes [59,60]. In conflict with these findings are conclusions from a Swedish study which followed disease progression in 900 patients for 13 years. High phospho-STAT3 levels were associated with lower risk of early recurrence, lower rates of distant metastases, lower histological grade, smaller tumor size and overall better prognosis [61]. This is corroborated by a meta-analysis and protein array data correlating nuclear phospho-STAT3 and STAT3 mRNA expression to more favorable outcomes for breast cancer patients [62]. It is unclear why there are differences in the conclusions drawn on the prognostic value of STAT3 in breast cancer. Differences in the patient cohorts such as their chemotherapy status, grade of disease and metastatic stage may be a factor, as could differences in staining technique and specificity of phospho-STAT3 assessment. Pathology assessment of phospho-STAT3 positive patients may be determined using a different standard for different studies, leading to conflicting conclusions being drawn. The prognostic significance of STAT3 remains unclear and warrants further investigation for its potential impact on therapeutic decisions.

## 3. STAT3 Activation in the Breast Cancer Metastatic Process

Preclinical studies provide functional evidence of STAT3 playing a critical role in the metastatic dissemination of breast cancer cells to distal sites, namely to bone, liver and lung [63]. Release of high levels of IL-11 from breast tumors is strongly correlated with an increased probability of developing bone metastases [64], while higher circulating serum levels of IL-6 are associated with the presence of >1 metastatic site and with liver metastasis, which occurs in advanced metastatic disease [65]. Cell signaling, induced by IL-11 and IL-6 and the downstream STAT3, may promote the breast cancer metastatic process by a wide range of molecular processes, and further studies that elaborate on the different tumor-promoting effects of IL-6 and IL-11 may lead to research into effective treatments for metastatic disease.

IL-11 has a significant role in the normal physiological function of the bone matrix microenvironment, with its receptor IL-11RA expressed on both osteoblasts and osteoclasts [66]. IL-11-induced STAT3 activity stimulates osteoblast activity, and accordingly transgenic mice overexpressing IL-11 showed increased bone formation [66]. IL-11 is also critical in stimulating the differentiation of osteoclasts via the inhibition of osteoprotegerin (OPG) and the promotion of nuclear factor ligand-receptor B (RANKL) [67] to tip the ratio in favor of bone resorption, with IL-11RA knockout animals having a low number of osteoclasts compared with wildtype littermates [68]. It appears to be osteoclastic actions which are dominant in the metastasis of breast cancer cells to the bone. Given IL-11 is known to play a significant role in the production of osteoclasts from progenitor cells in the bone marrow, the invasion of metastatic breast cancer cells commonly leads to bone destruction though the signaling actions of IL-11 and parathyroid hormone-related protein (PTHrP) released from tumor cells to activate osteoclasts [69]. The action of osteoclasts on bone remodeling releases TGF-β into the metastatic microenvironment which, in turn, stimulates the production of IL-11 and other osteoclast differentiating factors in breast cancer cells (Figure 2) [70,71]. This creates a positive feedback loop and further accelerates the rate of bone loss seen in patients with metastatic disease, worsening survival outcomes. Accordingly, serum levels of IL-11, STAT3 and TGF-β were higher in patients with metastases to the bone compared with patients with only primary breast cancer, with the metastatic group showing associated poorer survival outcomes [55]. This has been supported experimentally by a study by Liang et al. [56], who showed that BoM-1833 cells, a human breast cancer cell line which metastasizes specifically to the bone in nude mice, show higher osteolytic rates compared with estrogen receptor positive (ER+) MCF7 and the TNBC cell line MDA-MB-231. IL-11 is the key factor driving the increased formation of osteoclasts, through the STAT3 activation of c-MYC [72,73]. These osteolytic processes may also be mediated by miRNA or the lncRNA action on IL-11 expression. The lncRNA-ATB is upregulated by TGF-β to stabilize IL-11 transcripts in hepatocellular cancer cells, and its upregulation is also observed in early metastatic breast cancer cells to promote survival within the metastatic niche via IL-11/STAT3-dependent mechanisms [72]. Several miRNAs have been identified that may inhibit IL-11 in bone metastatic breast cancer cells, including miR-204, miR-211, miR-379 [74] and miR-124 [75], raising the possibility of miRNA-mimicking therapeutics to limit bone loss in metastatic breast cancer.

IL-6 is also known to promote breast cancer invasion to the bone, upregulating the chemokine receptor CXCR4 via STAT3 signaling in breast cells to promote proliferation of breast cells to the bone matrix [76]. However, IL-6 appears to promote metastasis primarily as an inflammatory factor capable of mediating epithelial-to-mesenchymal transition (EMT), a key phenotype of metastatic cancer cells. EMT is a reversible process that is characterized by a distinct set of genetic, biochemical and molecular changes resulting in tumor epithelial cells acquiring a more mesenchymal phenotype, which afford greater tumorigenic and metastatic potential [77]. Characteristic features of EMT which promote metastasis include loss of cell-to-cell junctions, loss of cell polarity, cytoskeletal reorganization, degradation of the basement membrane and reprogrammed gene expression [78]. IL-6 signaling via activated STAT3 is known to be a mediator of EMT processes in adamantinomatous craniopharyngioma—a subtype of brain cancer [79], cervical cancer [80], esophageal cancer [81], and bladder cancer [82], as well as in head and neck carcinoma [83]. Alongside TGFβ, IL-6 is one of the primary extracellular signals that initiates pro-EMT signaling pathways including JAK/STAT3, SMAD signaling and PI3K/AKT cascades [84]. Treatment of the ER+ breast cancer cell line MCF7 with IL-6 induces an EMT phenotype in vitro, resulting in the upregulation of key EMT genes such as N-cadherin, Snail, Vimentin and Twist, as well as, the loss of E-cadherin expression [85]. This enhances the invasive capacity of MCF7 cells into the extracellular matrix [86]. Co-culture of TNBC cell line MDA-MB-231 with primary human adipose stromal cells resulted in a more invasive and aggressive phenotype, an effect which was abrogated when IL-6 was depleted from the culture medium [18]. This demonstrates that IL-6-mediated EMT is not exclusive to a particular subtype of breast cancer. The primary source of EMT-activating IL-6 within the breast metastatic niche appears to be the mature adipocytes, which secrete IL-6 and stimulate STAT3 signaling in tumor epithelial cells to trigger EMT response, including enhanced cell migration and invasion downstream of IL-6/STAT3-stimulated lysyl hydroxylase-2 (LH2) and tropomyosin receptor kinase B (TrkB) expression [87,88,89]. This upregulation in IL-6 expression via NF-κB signaling in the breast tumor microenvironment leads to recruitment of neutrophils and myeloid-derived suppressor cells (MDSCs) to further promote tumor cell invasiveness and metastasis to the lung [90,91]. This elevated IL-6 secretion also promotes THP-1 monocyte polarization into M2-like macrophages, an event which drives the invasiveness of TNBC cells [92].

Molecular alterations leading to metastasis mediated by IL-6/STAT3 signaling appear to be through a hijacking of ERα enhancers in hormone receptor positive breast tumors. Although ERα and STAT3 are known to share enhancer activity mutually with FOXA1, in metastatic models of disease, STAT3 establishes shared enhancers independent of FOXA1. This drives distinct transcriptional programs from that of ERα and, as such, the JAK inhibitor Ruxolitinib reduces in vivo invasiveness of ER+ breast cancer models [93]. In hormone therapy-resistant breast cancers, decreased expression of ERα is observed, and found to be inversely correlated with increased circulating IL-6 levels and increased metastatic progression in breast cancer patients [94]. In Tamoxifen and Fulvestrant-resistant breast cancer, higher levels of secreted and autocrine IL-6 levels are measurable when compared with Tamoxifen-responsive tumors in both the primary tumors and the metastases [94]. Patient-derived Tamoxifen- and Fulvestrant-resistant metastatic lesions when implanted as xenografts, show substantially reduced growth in mice receiving a combination of hormone therapies with anti-IL-6 antibody, Tocilizumab, when compared with single-treatment metastases [94]. This sensitization to hormone therapy was attributed to reduced STAT3, Notch3 and CD133 expression in metastatic tumor cells. This study comprehensively demonstrated that suppression of IL-6 signaling sensitizes tumor cells to hormone therapies, suggestive of the potential therapeutic benefits of IL-6/STAT3 inhibition in luminal, hormone-dependent breast cancers.

## 4. Role of IL-6 Cytokine/STAT3 Signaling in Immune Evasion and Function in Metastatic Cancer

### 4.1. STAT3 Signaling and Immune Evasion

The impact of the dampening down of immune responses contributing to cancer development and progression is now well established. There are now a range of new immunotherapies, being developed to enhance checkpoint blockades and other endogenous anti-tumor immune responses. Apart from the antigens that the tumor cell may express, heterogeneity of cancer-associated fibroblast (CAF) phenotypes can influence the degree of immunosuppression, impacting cancer metastasis. Cytokine signaling is a major mechanism that mediates the crosstalk between different cell types within the TME to override the CD8^+^ cytotoxic and CD4^+^ helper T cell functions. A critical cytokine-dependent mechanism that promotes immune evasion during metastasis is transforming growth factor β (TGFβ)/IL-11 signaling axis [95,96]. Evidence for its’ functional role in metastasis was elegantly demonstrated in preclinical colorectal cancer (CRC) liver metastasis models, as well as in the profiling of CRC clinical samples, showing that the secretion of IL-11 by TGF-β-stimulated CAFs led to gp130/STAT3 signaling activation in the cells of the TME to mediate metastatic outgrowth in the liver [96]. Furthermore, high TGF-β/IL-11 expression signatures in the CAFs are associated with poorer patient-survival outcomes and a high risk of CRC relapse upon treatment. As treatment with a TGFBR1-specific inhibitor Galunisertib dramatically suppresses CRC liver metastasis, inhibitors for the IL-11/gp130/STAT3 pathways may also evoke similar anti-metastatic responses [36].

Interestingly, the combination of Galunisertib and an anti-PD-L1 antibody treatment induces a potent anti-tumor cytotoxic T cell response to prevent the metastatic colonization of tumor cells in the liver. Importantly, this combination treatment diminished metastatic disease in animal models with overt metastatic disease. The enhanced response to anti-PD-L1 antibody treatment, in combination with galunisertib highlights the importance of the TGFβ-dependent suppression of CD8^+^ T cell anti-tumor cytolytic activity. Although the relationship between TGFβ /IL-11 signaling was explored mainly in CAFs, the effects of IL-11 on mechanisms of immune evasion during cancer metastasis have not yet been demonstrated in any cancer context. Although, we have evaluated the role of IL-11 signaling on immune cell activity in primary tumors using CRC models to demonstrate a prominent role of IL-11 in mediating CD4^+^ T cell-dependent immune-evasion in the tumor microenvironment [97].

The direct cellular effect of STAT3 hyperactivation in tumor-infiltrating immune cells to evoke immunosuppression occurs via the inhibition of both innate and adaptive immune responses. An elevated STAT3 transcription program in innate immune cell subsets has been linked to the production of pro-inflammatory mediators such as IFNγ, dampened down antigen presentation, and the inhibition of cytolytic activities of effector cells. In adaptive immune cell subsets, elevated STAT3 activity causes decreased accumulation of effector T cells [3].

Through the activation of a STAT3 transcriptional program, genes involved in T cell activation and differentiation include retinoic acid-related orphan receptor-γ (Rorc), Malt1, Tigit, Ceacam1, Chd7, IL-21, Eomes and Rsad2 [98]. Furthermore, IL-6, placental growth factor (PlGF) and Cxxc finger protein 1 (Cxxc1) are known key upstream activators of STAT3 signaling in undifferentiated CD4^+^ T cells, promoting the polarization to Th17 cells. The expansion of myeloid-derived suppressor cells (MDSCs) in the tumor microenvironment is mediated by STAT3 [99,100] and the differentiation of tumor-associated macrophages (TAMs) [99]. Furthermore, the genetic ablation of Stat3 in the hematopoietic cells shows significant regression of primary tumor growth and the suppression of metastasis [100]. In these mice, the anti-metastatic responses observed in tumor-bearing mice with Stat3-deficient hematopoietic cells, correlated to the enhanced function of dendritic cells, T cells, natural killer (NK) cells and neutrophils [100] (Figure 2). These studies provide evidence of the requirement of STAT3 signaling in immune evasion mechanisms in a range of cell types within the tumor microenvironment; however, to date there is little known of the exact mechanism underpinning STAT3-dependent antitumor immunity in metastatic breast cancer, and thus, this merits further interrogation to determine its potential in enhancing cancer immunotherapies.

### 4.2. STAT3 and Macrophage Polarization

Tumor-associated macrophages (TAMs) contribute to the progression of advanced cancer and metastasis by evoking a multitude of changes in the tumor microenvironment, including matrix remodeling, angiogenesis, immunosuppression and mechanisms of treatment resistance [101,102]. Recent efforts have been directed at developing immune-cell-specific therapies, including those targeting TAMs [103]. Within the tumor microenvironment, the main cues that drive macrophage polarization from classically activated (M1) to alternatively activated (M2) include IL-4, IL-13, IL-34 and CSF-1 [103]. The activation states of TAMs are heterogeneous and reflective of cancer-type, stage and treatment exposure. Upon polarization to M2, TAMs produce pro-inflammatory cytokines, including IL-6 and TNFα which act in a pro-tumorigenic fashion [100]. STAT3 and STAT6 have been linked to M2 polarization in tumor progression [104], although there are studies that demonstrate that the specific loss of myeloid STAT3 activity enhances cancer progression. In the context of TNBC, the conditional deletion of STAT3 using the c-fms-iCre model increased mammary tumor incidence, an effect mediated by the induction of cyclooxygenase-2 (COX-2) [105]. This upregulation of COX2 in macrophages also occurred when the JAK inhibitor ruxolitinib was utilized as a therapy, indicating that changes in macrophage properties can influence the development of therapeutic resistance. In this particular study, it was further shown that the use of the COX-2 inhibitor, Celecoxib, enhanced the efficacy of Ruxolitinib in reducing mammary tumor growth, suggesting that combination therapies that target multiple factors within the tumor microenvironment is likely the most effective strategy in treating TNBC. STAT3 inhibition experiments show immune cell specific effects, as demonstrated in the Mx1-cre-mediated STAT3 deletion model, in which antigen presentation by dendritic cells was enhanced to promote anti-tumor T cell responses [100]. This has also been demonstrated with LysM-Cre–mediated STAT3 deletion which reduced tumor growth in pancreatic tumor models [104] and improved T cell-mediated anti-tumor responses observed in models of lung [106] and colorectal tumors [107]. Collectively, the findings of these studies implicate two key points: (i) the importance of understanding upstream cell signaling cues that would block STAT3 activation selectively in tumor, stromal or immune cells; and (ii) the effects of combination with additional pharmacological agents to overcome the deleterious “side effects” of STAT3 inhibition in a particular immune cell type, such as macrophages, as demonstrated with the combination of ruxolitinib and celecoxib in the TNBC model. Further studies in different cancers will provide important information on the value of targeting macrophage-dependent STAT3 activity. The blockade of IL-6 would serve to block the downstream, protumourigenic effects of TAMs, thus supporting the idea that targeting of IL-6R and gp130 may present a better approach to inhibition of this pathway in breast cancers instead of the use of JAK/STAT3 inhibitors.

## 5. Therapeutically Targeting IL-6 Signaling and STAT3 Activation in Breast Cancer

Although there is clear and mounting evidence for the role of IL-6 and IL-11-mediated STAT3 signaling in tumor establishment, proliferation, immune evasion and metastatic disease, to date there are no FDA-approved therapies which target the IL-6/IL-11/STAT3 axis for use in the treatment of any breast cancer subtype. However, many pre-clinical studies have highlighted the potential for such therapies, and a number of clinical trials are currently evaluating safety and efficacy of anti-IL-6/IL-11/STAT3 agents for the treatment of breast cancer. Treatment strategies fall into two broad categories: small molecule inhibitors of STAT3 signaling pathway components, and monoclonal antibodies directed against the cytokines or their receptors (Table 1).

### 5.1. Small-Molecule Inhibitor Therapies

Pre-clinical in vitro and in vivo studies have identified a number of candidate small molecule inhibitors that target the STAT3 signaling cascade, namely gp130, JAK and STAT3 itself, which inhibit growth and invasion of breast cancer models. A strong candidate for clinical use is the repurposed drug Bazedoxifene, a selective estrogen receptor modulator (SERM) currently approved for the treatment of osteoporosis, but which has also shown to disrupt protein–protein interactions between IL-6/IL-11 and the gp130 receptor [2,108,109]. Studies in animal models of spontaneous gastrointestinal cancer with hyperactive gp130 signaling, mediated via IL-11, show that Bazedoxifene treatment suppressed tumor growth via STAT3 mechanisms [2]. While some studies have suggested that Bazedoxifene alone or in combination with chemotherapy has anti-proliferative effects on ER+ and TNBC cells via STAT3 [110,111,112], there is limited pre-clinical evidence for its use as a breast cancer therapeutic in the context of excessive STAT3 signaling at the present time. However, some clinical trials have examined its use as a SERM for the prevention of cancer or as a therapy for advanced breast cancer (NCT02448771, NCT04821141). Safety data from these trials may inform further research into the future repurposing of Bazedoxifene as a therapeutic agent for STAT3-high tumors.

Likewise, the therapeutic targeting of cytokine receptor-associated JAKs has been approved for use in the treatment of rheumatoid arthritis, Crohn’s disease and other inflammatory diseases [3] but its applications in cancer are substantially more limited. One issue has been these early JAK inhibitors did not specifically distinguish between the four highly related JAK family members, thereby causing blanket inhibition and poor side effects. However, more recent developments in small-molecule JAK inhibitor specificity have allowed for more targeted approaches that may be further studied in the context of targeted breast cancer treatments. Amplification of *JAK2* at the 9p24 locus has been identified in a subset of triple negative breast cancers, and JAK1/2-specific inhibitors such as Baricitinib may be of most benefit to patients with these tumors [113,114]. The pre-clinical assessment of JAK2 inhibition in combination with SMO-GLI1/tGLI1 inhibitors demonstrated the synergistic inhibition of primary tumor growth and metastasis, prolonging survival in rodent breast cancer models [43]. A phase II clinical trial assessed the efficacy of the JAK1/2 inhibitor Ruxolitinib in metastatic TNBC; however, it was noted that active STAT3 signaling in these patients did not decline in response to treatment, and there was insufficient clinical benefit observed [115]. This data indicates that while there is potential for clinical benefit in JAK inhibition, in terms of reducing tumor growth, identifying patient’s specific molecular signature, in which JAK inhibition is an effective treatment strategy is required.

Finally, the direct inhibition of STAT3 through prevention of its phosphorylation or DNA binding is also a viable therapeutic option. Several compounds have been identified in pre-clinical laboratory studies as inhibiting STAT3 activity in cell culture or in vivo mice models, particularly in the context of TNBC where other treatment options are more limited. A non-peptide inhibitor LLY17 shows a capacity to inhibit proliferation and induce apoptosis in human and murine TNBC cells in vitro and in vivo [116]. Similar results have been demonstrated for pyrimethamine, a repurposed drug that is an approved antiparasitic, anti-malarial drug. Interestingly, tumor reduction in vivo with pyrimethamine treatment was mediated by enhanced infiltration of cytotoxic T-cells, suggestive of its effects on multiple cells within the tumor microenvironment [117]. This is supported by a study demonstrating that the inhibition of STAT3 in combination with STAT1 in TNBC results in the downregulation of PD-L1, a result which supports the development of STAT3/STAT1 inhibition as part of an immunotherapy approach [118]. More recently, in silico screens have identified a number of natural and synthetic molecules such as 15-Keto prostaglandin E2, Osthole and Betulinic acid have the capacity to inhibit STAT3 activity and, as a consequence, suppress breast cancer cell growth; however, these studies have not progressed beyond the in vitro assay phase [119,120,121]. There is limited clinical trial evidence to support the use of STAT3 inhibitors as a breast cancer therapeutic, although one current early-phase trial is currently evaluating the efficacy of an oral STAT3 inhibitor TTI-101 [122] for a range of late-stage cancers including breast cancer (NCT03195699). Whilst results for this study remain to be evaluated, there is much scope for the further exploration of small molecule STAT3 inhibition in a clinical breast cancer setting.

### 5.2. Monoclonal Antibodies

A second approach being explored pre-clinically and in early clinical trials is to block the cytokines IL-6/IL-11 or their receptors with monoclonal antibodies. The IL-6 or IL-6R mAbs, such as Clazakizumab and Tocilizumab, have been trialed clinically for the treatment of organ transplant rejection [123], rheumatoid arthritis [124] and COVID-19 (Table 1) [125,126]. In a cancer setting, the IL-6R mAb Tocilizumab inhibits the phosphorylation of STAT3 in osteosarcoma cell lines, leading to the attenuation of pro-growth signaling and resultant reduction in cell proliferation [127]. Similar results have also been observed in non-small-cell lung cancer cells [128]. IL-6 antibody treatment inhibits in vitro cell proliferation and in vivo tumor growth in both ER+ MCF7 cells and TNBC, MDA-MB-231 cells through a blockade of STAT3 activation and associated downregulation of STAT3 target genes including SOCS3, IL6ST and genes involved in angiogenesis [129,130]. Treatment with Tocilizumab in mouse models of hormone-therapy-resistant breast cancer can also re-sensitize tumors to Tamoxifen treatment, highlighting the importance of the IL-6-IL-6R-ER axis in hormone receptor positive disease [94]. The IL-6 mAb with the most clinical development is Siltuximab, which has been studied in early-phase clinical trials for the treatment of metastatic pancreatic cancer (NCT04191421), renal cell carcinoma, prostate cancer, and Castleman’s disease (a lymphoproliferative disorder) [131,132,133]. The blocking of the IL-6 receptor may also be a viable therapeutic option, showing in vivo capacity to attenuate STAT3 signaling in models of pancreatic ductal adenocarcinoma [134]. No clinical studies have been performed which examine the clinical utility of IL-6 or IL-6 receptor antibodies in a breast cancer setting; however, given the promising results from trials in other tumor streams, there is a clear potential for examining the antibody blockade for breast cancer patients.

There is limited research into antibodies that target IL-11 or its receptor in cancers. In an in vitro co-culture model of gastric cancer cells and cancer-associated fibroblasts where high IL-11 secretion was measured, treatment with an IL-11 neutralizing antibody significantly inhibited JAK/STAT3 activity leading to reduced cell migration and invasion [135]. There is also strong evidence for the use of IL-11 receptor blocking antibodies to attenuate STAT3-mediated proliferation in cell line and mouse models of endometrial cancer [136,137]. In the context of breast cancer, IL-11 neutralizing antibodies appear to downregulate MMP genes and upregulate EMT signatures in the triple negative MDA-MB-231 cell line [138]. Despite evidence for the substantial role of IL-11 in inducing STAT3 activity in breast cancer, there is limited research examining how its blockade may be exploited therapeutically.

### 5.3. Limitations to STAT3-Targeting Therapies

While there is clear potential for targeting STAT3 and its activating cytokines as a therapeutic strategy for breast cancer, limitations of this approach also need to be considered. Firstly, targeting IL-6 and its receptors could result in a decline in the ability of these signaling pathways to perform vital host immune defense and tissue regeneration functions [3]. One strategy used to overcome deleterious side effects of global IL-6 blockade, in order to specifically target chronic inflammation and tissue damage, is the use of a recombinant soluble gp130 receptor (soluble gp130Fc) Olamkicept, which inactivates trans-IL-6 signaling while retaining the classical IL-6 signaling functions which are most involved in mediating immune responses [139]. While this is yet to be trialed in cancer setting, it may present a promising option, especially in the treatment of lung and colorectal cancers [140]. Furthermore, disrupting the IL-11/IL11RA interaction is thought to have fewer off-target effects than IL-6 targeting, and its benefit has been shown pre-clinically in mouse models of gastrointestinal cancer [141].

The highly conserved nature of STAT family transcription factors could also result in upstream targeting having non-specific effects on other STAT-induced signaling cascades [142]. For example, STAT1 and STAT3 contain highly conserved regions and significant homology, leading to some off-target effects of the STAT3 blockade on STAT1 function [143]. Whilst STAT3 is thought to be significantly involved in tumor biology, STAT1 is thought to have a role in pathogen defense, growth inhibition and apoptosis [144]. Therefore, targeting STAT3 can inadvertently lead to the downregulation of STAT1 and the associated advantages to tumor cells [145].

Direct inhibitors of STAT3 that prevent nuclear translocation or the binding of STAT3 to DNA have been found to be non-specific in terms of their mechanism of action. To date, there has been limited efficacy demonstrated with single-agent STAT3 inhibitor therapy, Napabucasin (BBI608) in colorectal cancer patients (Clinical Trial NCT01830621 [146]). However, when evaluating patient outcomes in those with high phospho-STAT3 levels, responses to Napabucasin appeared to be beneficial. No major adverse side effects were reported in this trial. This data reflects that patient selection may be an important parameter to take into consideration. Furthermore, outcomes from the clinical trial of combination treatment of inhibitors of the STAT3 signaling pathway with chemo- and immuno-therapy in cancer patients will better inform of further clinical utility.

Finally, the direct targeting of STAT3 by inhibiting its phosphorylation without affecting its protein stability could have long term implications leading to compensatory mechanisms by other signaling pathways, ultimately leading to drug resistance [142]. This could include other sites of STAT3 phosphorylation or acetylation to confer active transcription [147,148] or through heterodimerization with STAT1 or STAT5 to form a DNA-binding complex [149,150]. Therefore, it may be of greater therapeutic benefit to investigate the potential for small-molecules which target STAT3 for ubiquitination and complete degradation. One such example is SD-36, a small-molecule inhibitor of STAT3 which results in its degradation and is able to cause strong regression of in vivo tumor growth in mouse leukemia and lymphoma models [151]. These results suggest that the further development of STAT3-degrading small molecules may be of therapeutic benefit.

## 6. Conclusions

The literature and clinical study evidence highlight a clear role for IL-6 and IL-11-induced STAT3 activation in driving pro-tumorigenic processes and metastatic dissemination in all types of breast cancer. The activation of the IL-6 and IL-11-dependent signaling cascade leads to the phosphorylation of STAT3 to transcriptionally activate genes and pathways that promote breast cancer proliferation, suppress apoptosis, allow for immune evasion leading to metastatic growth, and attain chemoresistance. Taken together, there is a vast array of evidence demonstrating STAT3 as a pro-tumorigenic transcription factor implicated in many independent aspects of tumor biology. Accordingly, highly active STAT3 signaling may be a negative prognostic indicator in breast cancer patients, although epidemiological evidence remains uncertain. STAT3 signaling is heavily involved in immune evasion and chemoresistance in breast cancer, and ongoing research in this field is leading to new advances in therapies for advanced, high-grade metastatic disease.

Based on our literature analysis, the therapeutic blockade of the IL-6/IL-11/gp130/STAT3 signaling axis presents an attractive yet challenging therapeutic option, either alone or in combination with other existing treatments such as chemotherapy or immunotherapy. Some small-molecule inhibitors or antibodies that target various components of the IL-6/IL-11-induced signaling pathway are already in routine clinical use for other indications, primarily inflammatory-related disorders. Further research and efficacy trials are required before they may be approved for use as a breast cancer therapy. For TNBC, in particular, there is an urgent clinical need to identify actionable targets, as cytotoxic chemotherapy and PD-L1 immunotherapy are the only currently approved treatment regimens. There is overwhelming evidence for the role of STAT3 in driving TNBC, and clinical trials to assess its blockade for TNBC patients are warranted. This may be contingent on the advent of personalized care approaches, where suitable patients with high STAT3 signaling may be identified through molecular profiling for STAT3 targeting therapies.

## Figures and Tables

**Table 1:** Small molecule inhibitors and antibodies targeting IL6/STAT3 signaling.

Therapeutic	Target	Mechanism	FDA Approval
Bazedoxifene	gp130 receptor	Selective estrogen receptor modulator (SERM) which disrupts interaction between IL6 and gp130	Menopause osteoporosis and moderate to severe hot flushes
Baricitinib	JAK1 and JAK2	Small molecule inhibition of JAK1 and JAK2	Rheumatoid arthritis
Ruxolitinib	JAK1 and JAK2	Small molecule inhibition of JAK1 and JAK2	Myelofibrosis and polycythemia vera
LLY17	STAT3	Inhibits STAT3 phosphorylation and dimerization	N/A
Pyrimethamine	STAT3	Inhibition of STAT3 Phosphorylation	N/A
TTI-01	STAT3	Inhibits recruitment of STAT3 to activated cytokine receptor complexes and dimerization	N/A
BBI608	STAT3	Inhibition of STAT3 Phosphorylation	N/A
SD-36	STAT3	Targets STAT3 for ubiquitination and proteasomal degradation	N/A
Clazakizumab	IL-6	Monoclonal antibody against IL6 ligand	N/A
Tocilizumab	IL-6R	Monoclonal antibody against IL6 receptor	Rheumatoid arthritis, systemic juvenile idiopathic arthritis, cytokine release syndrome, COVID-19
Siltuximab	IL-6	Monoclonal antibody against IL6 ligand	Idiopathic multicentric Castleman disease
